# Tackling Heterogeneity: A Leaf Disc-Based Assay for the High-Throughput Screening of Transient Gene Expression in Tobacco

**DOI:** 10.1371/journal.pone.0045803

**Published:** 2012-09-21

**Authors:** Natalia Piotrzkowski, Stefan Schillberg, Stefan Rasche

**Affiliations:** Fraunhofer Institute for Molecular Biology and Applied Ecology (IME), Aachen, Germany; University of Leeds, United Kingdom

## Abstract

Transient *Agrobacterium*-mediated gene expression assays for *Nicotiana tabacum* (*N. tabacum*) are frequently used because they facilitate the comparison of multiple expression constructs regarding their capacity for maximum recombinant protein production. However, for three model proteins, we found that recombinant protein accumulation (rpa) was significantly influenced by leaf age and leaf position effects. The ratio between the highest and lowest amount of protein accumulation (max/min ratio) was found to be as high as 11. Therefore, construct-based impacts on the rpa level that are less than 11-fold will be masked by background noise. To address this problem, we developed a leaf disc-based screening assay and infiltration device that allows the rpa level in a whole tobacco plant to be reliably and reproducibly determined. The prototype of the leaf disc infiltration device allows 14 *Agrobacterium*-mediated infiltration events to be conducted in parallel. As shown for three model proteins, the average max/min rpa ratio was reduced to 1.4 using this method, which allows for a sensitive comparison of different genetic elements affecting recombinant protein expression.

## Introduction

*Nicotiana tabacum* is a well-characterized platform for the production of a wide range of recombinant proteins, such as antibodies, vaccines and biopharmaceuticals [1]. Although plant-based production platforms have many advantages, including the capacity for complex posttranslational modifications, cost effectiveness and excellent scale-up potential [2]–[4], they usually achieve only low levels of expression [5]. In the case of *N. tabacum*, typical expression levels are in the range of 1–2% of the total soluble protein (TSP). Expression levels are influenced by the transgene construct (for example, its promoter, UTR and subcellular localization) and general features of the recombinant protein (such as its size, stability, solubility and toxicity to the host) [6]–[8]. To obtain maximal yields of functional protein, transient *Agrobacterium*-mediated expression assays for *N. tabacum* are frequently used to determine the effects of vector elements on certain traits, such as recombinant protein accumulation (rpa) and protein stability. However, transient gene expression in tobacco has been reported to result in inhomogeneous protein accumulation. Many different factors are thought to be involved in this observation, such as the availability of phytohormones [9], [10], sugars [11], [12] and phenolic compounds [13], [14]; the presence of cell wall proteins affecting bacterial motion [15], [16]; uneven infiltration [17]; gene silencing [18]–[20]; a general incompatibility between *Agrobacterium* and the plant [21]; and leaf tissue senescence [22]–[26]. In transient expression assays, which are used to compare different expression constructs, factors such as the availability of hormones or sugars affect all transformation events in the same way and are therefore considered to have a only a weak effect on inhomogeneous protein accumulation. As different parts of a leaf or of a single plant are used for those assays, we expected leaf senescence and leaf position to have a stronger impact on inhomogenious protein accumulation than the other factors listed above.

We assessed the influences of the growth stage and leaf position on the rpa levels of three model proteins and found that both leaf age and the position within the leaf have significant impacts. To control for such heterogeneous protein accumulation, infiltrating and subsequently extracting the TSP from all the leaves of an entire tobacco plant would be necessary. Using this method, only a single construct could be analyzed per plant, and the screening of multiple constructs would be a time-consuming and laborious procedure. We hypothesize that a sufficiently precise measurement of the rpa can be obtained via the analysis of only a few randomly chosen plant parts rather than the analysis of an entire plant.

Based on this hypothesis, we developed a high-throughput leaf disc-based infiltration assay that allows for the identification of expression constructs that result in the highest rpa and protein stability for the efficient production of recombinant proteins.

**Figure 1:**
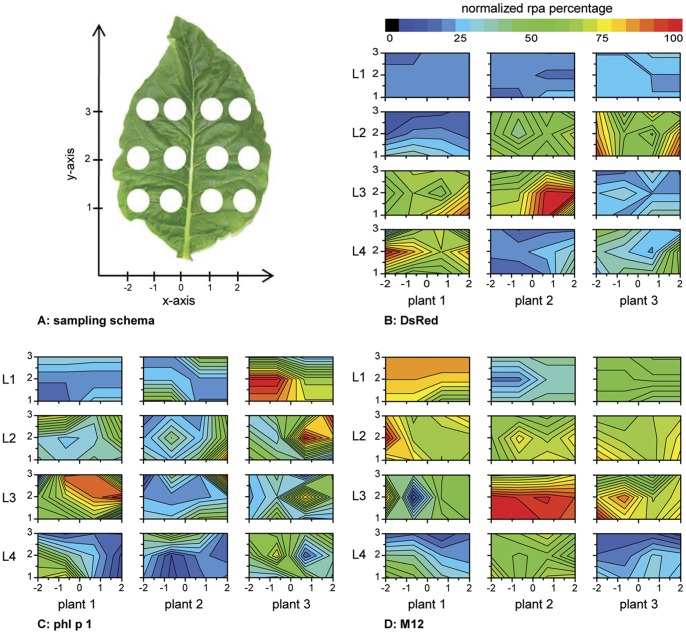
Distribution of DsRed, phl p 1 and M12 accumulation in vacuum-infiltrated tobacco leaves. Four leaves of various ages from eight-week-old *N. tabacum* plants were vacuum-infiltrated with *Agrobacterium* containing a plasmid encoding one of three model proteins: DsRed, phl p 1 or M12. After incubation (5 d, 25°C, 16/8 h light/dark cycle, 7 klux), the leaf discs were cut using a cork borer (22-mm inner diameter) according to the schema shown in Figure 1A. Figures 1B–D show the protein accumulation distribution for each of the three proteins with respect to the leaf age and position within the leaf. To simplify the evaluation and allow for a graphical illustration of the results, missing values between the data points were added by linear interpolation using Origin 8.1. For each of the three replicates, the four boxes designated L1 to L4 represent the four different leaf ages, with L4 being the oldest and L1 being the youngest. The notations 1–3 (y-axis) and −2−2 (x-axis) are the positional coordinates of the leaf discs (see Figure 1A). The level of rpa was related to the TSP to control for differences in leaf weight and extraction efficiency between individual leaf discs. The results obtained from each plant were normalized, with the highest rpa value set at 100%. The color scale represents the normalized rpa percentage.

## Results and Discussion

### Analysis of the Inhomogeneity of Recombinant Protein Accumulation in Transiently Transformed Tobacco Leaves

Three different proteins, the red fluorescent protein DsRed from *Discosoma strita* [27], the major timothy grass pollen allergen phl p 1 [28] and the recombinant human antibody M12 [29], were used to demonstrate the inhomogeneity of rpa in transiently transformed tobacco plants. We choose these proteins as models because they are representative of the diversity of recombinant proteins that are expressed in tobacco.

**Table 1:** Max/min ratios.

Protein	DsRed			phl p 1			M12		
SR1 plant	1	2	3	1	2	3	1	2	3
**Max/min ratio**	13.1	10.5	7.9	11.5	8.7	12.9	16.8	6.8	11.9
**Average max/min ratio**	10.5±2.6			11.0±2.1			11.8±5.0		

To obtain further information about the impact of the leaf age and leaf position on the rpa levels, the ratio of the highest to the lowest protein accumulation (max/min ratio) measured for each plant was determined for DsRed, phl p 1 and M12 (Figure 1).

Four leaves of various ages from an eight-week-old tobacco plant were vacuum-filtered with *A. tumefaciens* (GV3101) carrying the binary expression vector pTRA, which contained a gene for one of the three model proteins. After incubation, leaf discs were cut according to the schema shown in Figure 1A to assess the impacts of leaf age and position within the leaf on the rpa levels. Following extraction, the TSP was determined using the Bradford assay, and the recombinant protein concentration was determined with an ELISA (phl p 1, M12) or a fluorescence measurement (DsRed). The entire procedure was repeated twice for each model protein. To illustrate the inhomogeneity of the rpa levels, we summarized the results in the contour plots shown in Figure 1B–D. As shown by the graphs, the protein accumulation varied substantially between biological replicates and depending on the leaf age and position within a single leaf. The average ratio between the highest and lowest accumulation measured for each protein (max/min ratio) was approximately 11 (Table 1). Therefore, the construct-based impacts on the rpa levels will be masked by background noise until an 11-fold or greater difference is achieved. There are several examples in which expression constructs have been optimized to improve the yield of a target protein. These approaches lead to a 1.2–12-fold increase in rpa (Table 2), but because different transformation methods were used (e.g., protoplast transformation, *Agrobacterium*-mediated transformation or microprojectile bombardment), comparing or evaluating these results is difficult. However, these values give an idea of the levels that can be achieved when optimizing the expression constructs. Because almost all values are below the background noise of the conventional *Agrobacterium*-mediated transient screening assay, potentially useful constructs would have been lost during the screening procedure. In this case, the implementation of the leaf disc-based screening assay would be an advantage.

**Table 2:** Examples of successful expression construct optimization.

Modification	Host	Target protein	rpa improvement	Reference
Promoter	Tobacco protoplasts	GUS	4-fold	[35]
Leader sequence	Tobacco	GUS A	8.6-fold	[36]
Scaffold attachment region	Tobacco suspension culture	GUS	12-fold	[37]
Localization	Tobacco	LT-B*	1.2–2.8-fold	[38]
Fusion protein	Tobacco	GFP	2-fold	[39]
Localization	Tobacco	Antibody 14D9	2-fold	[40]

This table gives several examples for which the accumulation level of the target protein was improved by modifying the expression strategy.

* *E. coli* heat-labile enterotoxin B subunit (LT-B).

To assess the significance of leaf age and position within the leaf more precisely, we subjected the data to statistical analysis. A response surface model based on the existing data was set up using Design Experts 8.0.4 (historical data analysis). An analysis of variance (ANOVA) showed that, of the main factors, the leaf age and position within the leaf had a significant impact on the rpa level and that leaf age had the strongest effect (Table 3). Moreover, two interactions between the leaf age and position within the leaf (y-axis and x-axis) were revealed to be significant. Various combinations of the main factors and factor interactions had significant impacts on the rpa levels for each of the three proteins.

The predicted R^2^ values shown in Table 3 represent the predictive power of the model: the closer the values are to 1, the better the quality of the model, and lower values are an indication that missing factors have an impact on the response. All data were analyzed using the historical data analysis mode, and therefore, the predicted R^2^ values are not expected to be very high, as the data were not been collected according to a predefined model. All observations made for M12 and DsRed can be explained with the model. Only the low predicted R^2^ determined for phl p 1 indicates that one or more additional factors are involved. However, the ANOVA shows clearly that the leaf age and position within the leaf have a significant impact on the accumulation of all model proteins despite a missing factor involved in the phl p 1 accumulation. This result creates a heterogeneously distributed rpa, making the reliable comparison of different expression constructs difficult.

**Table 3:** ANOVA results.

Factors/Terms	DsRed	phl p 1	M12
**A**	−	0.2954	0.3097
**B**	0.0023	−	<0.0001
**C**	<0.0001	<0.0001	<0.0001
**AB**	−	−	0.059
**AC**	−	0.0002	−
**BC**	−	−	<0.0001
			
			
**R^2^**	0.7971	0.6588	0.9363
**Adj R^2^**	0.7751	0.5864	0.9184
**Pred R^2^**	0.7406	0.4637	0.8952

The rpa distribution data (Figure 1) were further analyzed with Design Experts 8.0.4 using the historical data analysis mode. The biological replicates for each model point were pooled before the analysis. In all cases, a 2FI model was used. The p-value for each model was less than 0.0001, indicating that the selected models were significant. Non-significant factors are not shown in this table. Factors: A = x-axis (−2, −1, 1, 2); B = y-axis (1, 2, 3); C = leaf age (L1, L2, L3, L4).

The infiltration of a whole tobacco plant and the extraction of the TSP from all leaves would homogenize such effects, but this method would be a time-consuming way to compare multiple expression constructs because only one construct could be analyzed per plant. Therefore, we investigated whether a defined number of randomly removed parts of a plant would produce a reliable estimate of the rpa level so that only these parts could be used for subsequent transient gene expression assays.

**Figure 2:**
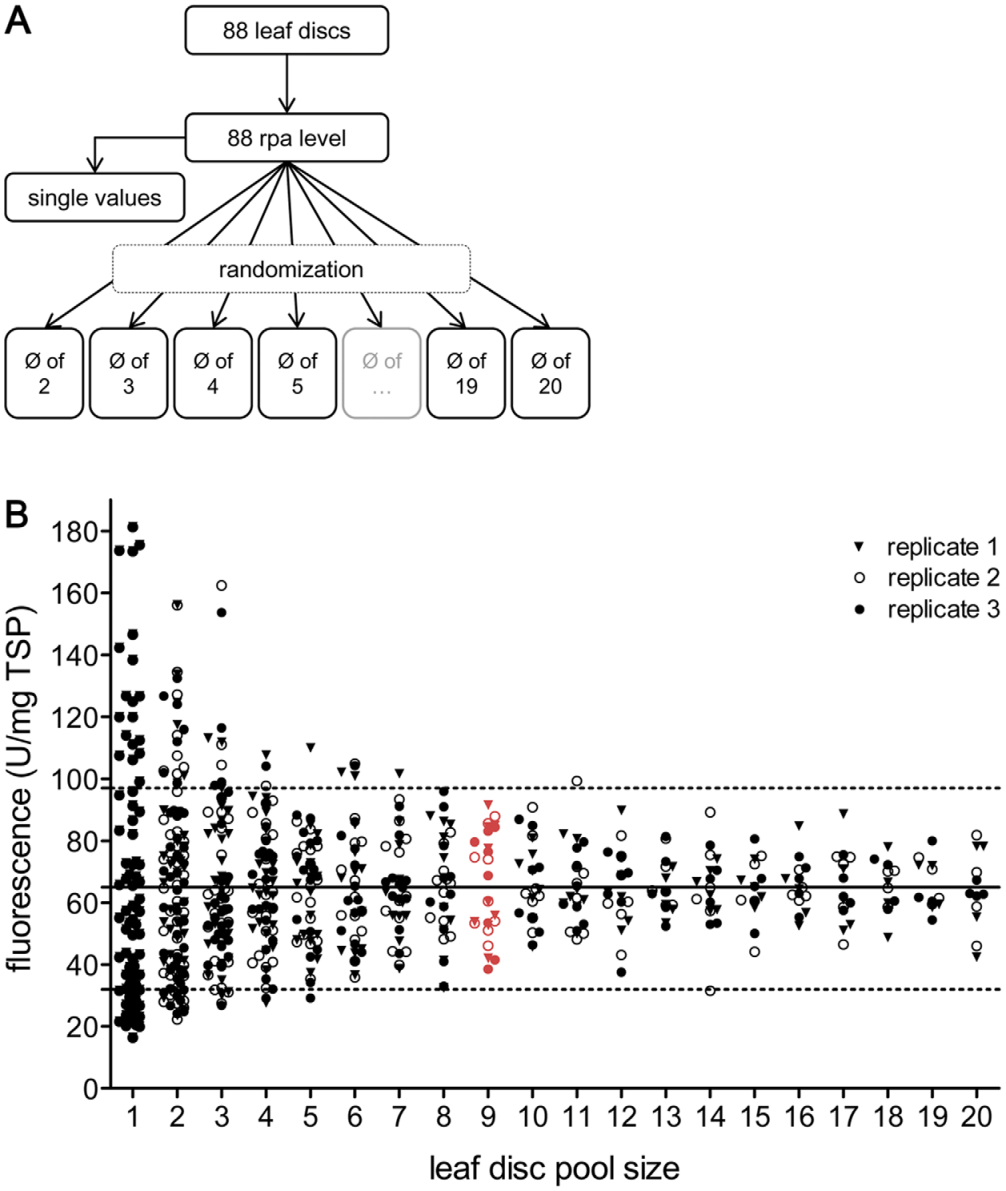
DsRed accumulation in the pools of multiple leaf discs. Leaf discs taken from all leaves of an eight-week-old *N. tabacum* plant were infiltrated with *Agrobacterium* containing a plasmid encoding DsRed. After a five-day incubation period (25°C, 16/8 h light/dark cycle, 7 klux), the TSP was extracted from 88 randomly selected leaf discs (Figure 2A). The DsRed accumulation was determined in each individual leaf disc extract by measuring its fluorescence, and this result was related to the TSP, which was determined via the Bradford assay. All values were randomly combined to generate virtual pools of 2, 3, 4 and so on, up to 20 leaf discs. The whole procedure was repeated twice for each pool. The outcome of this virtual pooling is shown in a scatter plot (Figure 2B) wherein each symbol represents one pool. To find the optimal pool size to minimize variation and effort, two constraints were set at 50% deviation (dotted lines) around the average fluorescence of all values (solid line). The selected pool size for all further assays (nine leaf discs) is indicated in red.

### Leaf Disc Assay

Briefly, 120 leaf discs were obtained from all leaves of an eight-week-old *N. tabacum* plant. The leaf discs were carefully randomized and vacuum-infiltrated using *A. tumefaciens* containing the plasmid encoding DsRed. After infiltration, the 88 most infiltrated discs (entirely translucent) were selected and incubated for five days. Subsequently, the TSP was extracted from each individual disc. The TSP concentration was calculated using the Bradford assay, and the DsRed accumulation was measured based on the fluorescence level, which was compared with a standard.

To determine the number of leaf discs necessary to achieve a uniform and reproducible result corresponding to the accumulation level in the whole plant, the DsRed values of the 88 single leaf discs were virtually pooled into groups of 2, 3, 4 and so on, up to 20 leaf discs (Figure 2A). Before generating these pools, all values were randomized to avoid systematical errors. Importantly, the accumulation levels calculated from single leaf discs or from pools of two or three discs showed a high level of variation between the individual values. This variation was caused by the different positions of the discs within the leaves and therefore by the age and quality of the leaf material. Pooling more leaf discs reduced the variation in the recombinant protein levels. According to Figure 2B, a set of at least nine leaf discs should be sufficient to provide a uniform value for the rpa level. Analyzing more than nine leaf discs would result in a slightly more precise value for the rpa while simultaneously increasing the labor input. Taking these factors into account, we decided to use nine leaf discs per infiltration.

**Table 4:** Leaf disc assay.

Protein	DsRed			phl p 1			M12		
Replicates	1	2	3	1	2	3	1	2	3
**Concentration (µg/mg TSP)**	6.9	5.6	5.7	11.7	11.0	11.6	17.4	9.0	17.3
**Average**	6.1±0.7			11.1±0.4			14.6±4.8		
**Max/min ratio**	1.2			1.1			1.9		

Three sets of twelve leaf discs each were infiltrated using *Agrobacterium* containing a construct encoding one of the three model proteins, DsRed, phl p 1 or M12. After the infiltration and a five-day incubation period (25°C, 16/8 h light/dark cycle, 7 klux), nine leaf discs from each set were pooled and extracted simultaneously. For phl p 1 and M12, the recombinant protein concentrations were determined via ELISA. The DsRed concentration was determined by measuring the fluorescence in the cell extract. All values were normalized to the TSP content, which was determined via Bradford assay. The max/min ratio indicates the difference between the highest and lowest concentration measured for each model protein.

To facilitate the analysis of a large number of different constructs and/or conditions, we designed a prototype leaf disc infiltration device to facilitate the parallel infiltration of 168 leaf discs in 14 sets (see figure S1).

To verify the assumption that a set of nine leaf discs is sufficient to provide a reliable estimate of the rpa level in a whole tobacco plant, we infiltrated three sets of leaf discs with *A. tumefaciens* harboring a construct encoding one of the three model proteins. The results clearly show that the leaf disc assay (LDA) provides uniform protein accumulation levels, resulting in a reduced max/min ratio of 1.1–1.9 (Table 4).

**Figure 3:**
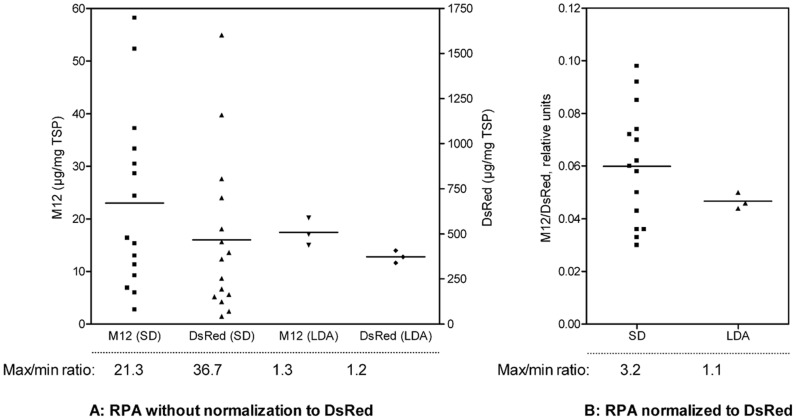
Comparison between leaf disc assay and the utilization of an internal marker. Randomly selected leaf discs from an eight-week-old *N. tabacum* plant were infiltrated with *A. tumefaciens* carrying a plasmid containing the expression cassettes for M12 (target protein) and DsRed (internal marker) on the same T-DNA. After incubation (5 d, 25°C, 16/8 h light/dark cycle, 7 klux), the TSP was extracted from 15 single leaf discs (SD) and from three sets of nine pooled leaf discs according to the leaf disc assay protocol (LDA). The M12 accumulation was determined via ELISA, and the DsRed concentration was determined by measuring the fluorescence. All values were related to the TSP determined via the Bradford assay and are represented by dots that vary around the average rpa level (Figure 3A). The max/min ratio between each individual leaf disc and the pooled discs are shown below the graph. The M12 accumulation normalized to the DsRed accumulation from the same leaf disc or pool of leaf discs are shown in Figure 3B.

### Leaf Disc Assay or Internal Marker

As an alternative approach to the LDA, a marker protein encoded on the same T-DNA as the target protein can be used to compensate for the experimental variation by normalizing the accumulation of the target protein to the accumulation of the internal marker [30]. To compare both methods in terms of reproducibility, accuracy and efficiency, we infiltrated 42 randomly selected leaf discs from an eight-week-old tobacco plant with *A. tumefaciens* carrying the binary expression vector pTRA, which contained the expression cassettes encoding the M12 antibody heavy and light chains and DsRed on the same T-DNA, with DsRed serving as an internal marker.

After a five-day incubation period, we extracted the TSP from all leaf discs and pooled 27 discs in three sets of nine discs each (according to the LDA). The remaining 15 discs were analyzed separately. Following the extraction, the TSP was determined using the Bradford assay. The M12 concentration was determined via ELISA, and the DsRed concentration by measuring the fluorescence.

**Table 5:** Construct screening.

	Apoplast	ER	Vacuole
**DsRed [µg/mg TSP]**	2.1±1.0	1.3±0.3	16.8±7.8
**Phl p 1 [µg/mg TSP]**	4.1±1.8	3.3±2.1	41.6±21.0
**M12 [µg/mg TSP]**	43.9±3.6	14.9±10.6	−

Three sets of 12 leaf discs each were infiltrated using *Agrobacterium* containing a construct coding for one of the eight constructs. After infiltration and a five-day incubation period (25°C, 16/8 h light/dark cycle, 7 klux), nine leaf discs from each set were pooled and extracted simultaneously. For phl p 1 and M12, the recombinant protein concentrations were determined via ELISA. The DsRed concentration was determined by measuring the fluorescence. All values were normalized to the TSP content as determined via Bradford assay. The max/min ratio indicates the difference between the highest and lowest concentration measured for each model protein.

Without normalization, the M12 and DsRed rpa levels varied strongly between the single leaf discs, resulting in a max/min ratio of 21.3 for M12 and 36.7 for DsRed (Figure 3A). In contrast, the rpa level determined from the pooled discs, which were analyzed according to the LDA protocol, showed only a max/min ration of 1.3 for M12 and 1.2 for DsRed. When normalizing the M12 accumulation to the accumulation of DsRed in the same leaf disc (Figure 3B), the max/min ratio was reduced to 3.2 for the single discs and to 1.1 for the pooled discs as determined by the LDA.

The use of an internal marker resulted in a significant reduction in the variance of individual measurements. The variance of the measurements according to the LDA method was slightly lower than those of the single values normalized to an internal marker. Both methods are suitable to reduce the variances of rpa levels, thereby improving the quality of the measurements. In our opinion, the LDA is simpler to apply than using an internal marker because there is no need to introduce an additional expression cassette containing the gene encoding the internal marker and regulatory elements into an existing expression vector. Furthermore, the quantification of an additional recombinant protein is inapplicable, reducing the probability of measurement errors.

### Construct Screening

To demonstrate that the LDA can be used to compare and optimize different expression constructs, we addressed whether the subcellular localization of each of the three model proteins would affect their accumulation levels. DsRed and phl p 1 were fused to signal peptide sequences to facilitate localization to the ER, apoplast or vacuole, whereas the M12 antibody was only targeted to the ER or apoplast. These sequences were cloned into the pTRA expression vector. After transformation into *A. tumefaciens*, transient transformation of *N. tabacum* was performed using the LDA as described above. The entire experiment was repeated twice (Table 5). For all three model proteins, 3–12-fold differences in the rpa level could be detected. For DsRed and phl p 1, the best results were obtained when targeting the proteins into the vacuole, whereas the highest accumulation levels for the M12 antibody were obtained in the apoplast.

### Conclusion

In this study, we have shown that the rpa level in transiently *Agrobacterium*-transformed tobacco leaves is significantly influenced by both the leaf age and position within the leaf. Because this variability negatively affects the outcome of transient screening assays, we developed a new, reproducible and reliable screening method based on the infiltration of randomly selected leaf discs obtained from all of the leaves of an adult tobacco plant. A set of nine leaf discs was sufficient to provide an adequate measurement of the rpa level of an entire tobacco plant, which was shown to be the case for three independent model proteins. Using this assay, the average max/min ratio of the rpa level was reduced from 11.1 (Table 1) to 1.4 (Table 4).The leaf disc-based infiltration approach allows different traits to be compared simultaneously on a small scale, thereby eliminating the primary drawback of conventional transient infiltration, which is inhomogeneous protein accumulation. As an example, we analyzed the impact of subcellular localization on the rpa of three model proteins, which resulted in a 3–12-fold increase in accumulation. Using a conventional screening approach, these optimized constructs would have been potentially eliminated due to high background noise.

## Materials and Methods

### Construction of Plant Expression Vectors and Cultivation of *Agrobacterium*

DNA encoding the model proteins DsRed [27] and phl p 1 [28] and the recombinant antibody M12 [29] were introduced into the plant expression vector pTRA, a derivative of pPAM (GenBank accession AY027531) following standard molecular cloning techniques. The sequence of the epitope tag, tag54, was c-terminally fused to the gene encoding phl p 1 to enable its detection via the tag54-specific monoclonal antibody mab54 [31]. The constructs used in the present study were introduced into *A. tumefaciens* GV3101 using a Multiporator (Eppendorf, Hamburg Germany) according to the manufacturer's instructions (Protocol No. 4308 915.502–12/2001). Successfully transformed *Agrobacteria* were stored at −80°C in 50% (v/v) glycerol.

To prepare the bacterial suspension for vacuum-mediated infiltration, 20 ml of YEB medium (0.5% [w/v] nutrient broth, 0.1% [w/v] yeast extract, 0.5% [w/v] tryptone, 0.5% [w/v] sucrose, pH 7.4, 2 mM MgSO_4_, 50 µg/ml kanamycin, 50 µg/ml rifampicin and 100 µg/ml carbenicillin) was inoculated with 500 µl of the *Agrobacterium* stock solution and incubated overnight at 28°C with shaking at 160 rpm. The next day, 300 ml of YEB medium supplemented with 10 mM glucose, 10 mM MES and 20 µM acetosyringone was inoculated with 10 ml of the pre-culture and incubated overnight at 28°C with shaking at 160 rpm. After incubation, the culture was adjusted to OD_600_ = 1 with 2× infiltration medium (10% [w/v] sucrose, 0.36% [w/v] glucose and 0.86% [w/v] MS basal salt mixture, pH 5.6) and water. Acetosyringone was added to a final concentration of 200 µM. Before infiltration, the mixture was incubated for 1 h at room temperature without shaking.

### *Agrobacterium*-mediated Transient Transformation

#### Infiltration of detached leaves

Leaves from eight-week-old *N. tabacum* plants were vacuum infiltrated with *Agrobacterium* as described by Kapila *et al.* [32]. After infiltration, the leaves were incubated on moist paper in a sealed tray for five days (25°C, 16/8 h light/dark cycle, 7 klux).

#### Infiltration of leaf discs

The leaf discs were removed from all leaves of eight-week-old *N. tabacum* plants using a 20-mm cork borer. All discs were gently mixed in a beaker filled with water. For each infiltration event, twelve randomly selected leaf discs were fixed in a custom-made leaf disc holder and positioned in an infiltration tank containing 120 ml of *Agrobacterium* solution. Up to 14 infiltration tanks could be subjected to vacuum infiltration (50 mbar, 2×20 min) in parallel. After infiltration, the leaf disc holder (including the leaf discs) was washed with water, and the nine most infiltrated leaf discs (entirely translucent) were selected and incubated for five days (25°C, 16/8 h light/dark cycle, 7 klux) in Petri dishes containing water-agar (1.5% [w/v] agar).

### Protein Extraction

The TSP was extracted from the infiltrated leaf discs with a motor-driven pestle using 3× (v/w) PBS as an extraction buffer. After extraction, the cell debris was removed by centrifugation (20 min, 13,000 g, 25°C). The remaining supernatant was stored on ice until use.

### Determination of Protein Accumulation Levels

To account for differences based on leaf weight, water content and extraction efficiency, all protein accumulation data were normalized to 1 mg TSP per ml extract as determined via the Bradford assay [33]. The protein concentrations were determined using competition ELISA (phl p 1), antibody quantification ELISA (M12) or by measuring the fluorescence (DsRed).

#### Competition ELISA

All incubation steps were performed for 1 h at 25°C. All peptides, antibodies and proteins were dissolved in PBS, and all dilution steps were performed using PBS. A high-binding ELISA plate was coated with 100 µl of streptavidin (1 mg/ml). Following incubation, all wells were blocked for 1 h by adding 100 µl of BSA (2% [w/v]). After three wash steps with 200 µl of PBS containing 0.05% (v/v) Tween 20 (PBS-T), 100 µl of biotinylated tag54-peptide (40 pmol/ml) was added to each well. After incubation, the peptide was discarded, and the plate was washed again three times with PBS-T.

Serial dilutions of the sample (1∶1–1∶1024) and a standard tag54 peptide (KHIKDWEHLEEF, 155–0.3 pmol/ml; Genscript, USA) were prepared in a separate round bottom plate (100 µl/well). To each well, 100 µl of the tag54-specific antibody mAb54 (0.5 µg/ml) was added and carefully mixed. From this mixture, 100 µl was transferred into the high-binding plate and incubated. After washing, 100 µl of an HRPO-labeled detection antibody (goat anti-mouse Fc specific serum, 0.16 µg/ml; Jackson ImmunoResearch, Newmarket, UK) was added to each well. The detection was performed by adding 100 µl of a 1 mg/ml 2, 20-azino-bis(3-ethyl benzthiazoline-6-sulfonic acid) (ABTS) substrate solution (Roche Applied Science, Mannheim, Germany), and the ELISA readings were recorded at 405 nm after incubation. The data were evaluated with Graph Pad Prism 4.0 (GraphPad Software, Inc.; La Jolla USA) using a sigmoidal dose-response equation with a variable slope.

#### Antibody quantification ELISA

The recombinant antibody M12 (kindly provided by Dr. Nicole Raven, Fraunhofer IME, Aachen, Germany) was quantified according to the protocol described by Raven *et al.* [34]. Briefly, microtiter plates (Greiner, Solingen Germany) were coated with 100 µl of goat anti-human IgG Fc fragment (Jackson ImmunoResearch)-specific serum in sodium bicarbonate buffer (pH 9.6) at 4°C for 16–24 hours. This serum was replaced with 200 µl per well of phosphate saline buffer containing 1% (w/v) BSA at room temperature for 1 h. The blocking buffer was removed, and 100 µl of each sample (serially diluted in PBS) was added to the ELISA plate and incubated at room temperature for 2 h. A protein A affinity chromatography-purified human IgG standard was also applied to serve as a reference for determining the M12 antibody concentration in the samples. Subsequently, the microtiter plates were washed with PBS containing 0.05% (v/v) Tween 20, and 100 µl of 0.2 µg/ml AP-labeled goat anti-human λ LC specific serum (Sigma-Aldrich, Deisenhofen, Germany) diluted in PBS was added to each well. After incubation at room temperature for 1 h, the wells were washed, and the detection was performed by adding 100 µl of para-nitrophenylphosphate (pNPP) substrate to each well. After incubation at room temperature for 30–50 min, the absorbance was measured at 405 nm.

#### Measurement of fluorescence

DsRed accumulation was determined by measuring the fluorescence (excitation 530/23 nm, emission 590/35 nm) of the plant extracts relative to a DsRed standard. Serial dilutions of each protein extract and of the standard (initial concentration 10 µg/ml) were prepared in PBS and measured in a black 96-well plate.

### Design of Experiments (DoE)

The statistical analysis of the protein accumulation data was performed using the historical data analysis mode of Design Experts 8.0.4 (Stat-Ease Inc., Minneapolis, USA). This mode allows for the evaluation of existing data and is capable of working with both numerical and categorical factors. Factor A: leaf x-axis, a numerical factor with four levels (−2, −1, 1, 2; Figure 1A). Factor B: leaf y-axis, a numerical factor with three levels (1, 2, 3; Figure 1A). Factor C: leaf age, a categorical factor with four levels (L1, L2, L3, L4). To simplify the analysis, we pooled the data from the three biological replicates for each protein and calculated the average for each model point. For all proteins, the software suggested a linear evaluation model, whereas non-significant terms were removed using backward selection. Due to the large number of model points, none of the analyzed terms were aliased with other terms.

## Supporting Information

Figure S1 **Leaf disc infiltration device.** The leaf disc holder, in which up to six individual leaf discs can be placed between the spacer bars fixed on the steel grid, is shown in Figure A. A second grid is placed on top of the first one to restrict the movement of the leaf discs. Two disc holders are placed in one infiltration tank (A, back) filled with the infiltration solution. Up to 14 tanks can be placed in the infiltration device (B), which is then subjected to a vacuum.
(TIF)
