# Supplementary figures and images for: Tackling Heterogeneity: A Leaf Disc-Based Assay for the High-Throughput Screening of Transient Gene Expression in Tobacco
Source: PLoS One. 2012 Sep 21;7(9):e45803. doi: 10.1371/journal.pone.0045803 (PMC3448687; doi:10.1371/journal.pone.0045803)

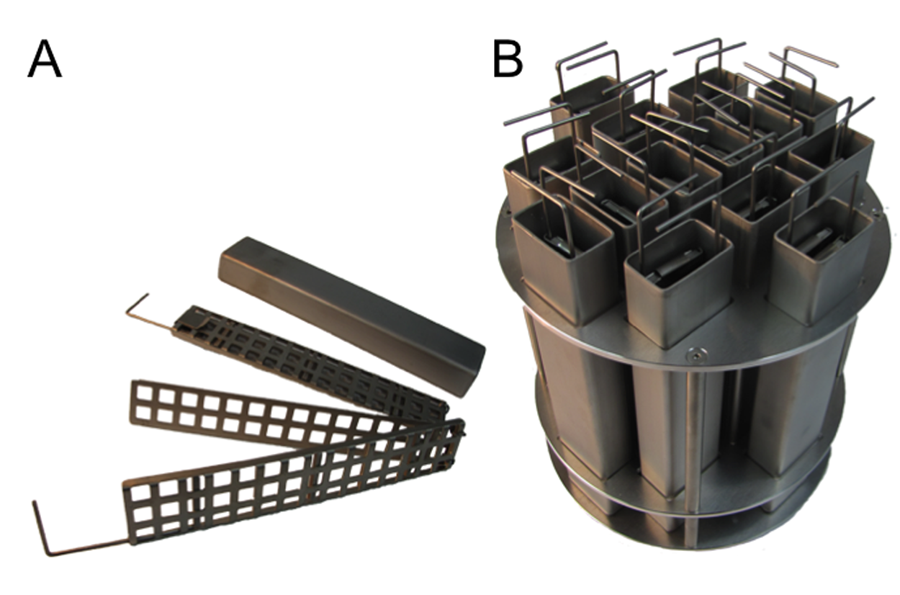

Supplement: Figure S1 — Leaf disc infiltration device. The leaf disc holder, in which up to six individual leaf discs can be placed between the spacer bars fixed on the steel grid, is shown in Figure A. A second grid is placed on top of the first one to restrict the movement of the leaf discs. Two disc holders are placed in one infiltration tank (A, back) filled with the infiltration solution. Up to 14 tanks can be placed in the infiltration device (B), which is then subjected to a vacuum. (TIF) [file pone.0045803.s001.tif]
